# Acute Phase Response and Neutrophils : Lymphocyte Ratio in Response to Astaxanthin in Staphylococcal Mice Mastitis Model

**DOI:** 10.1155/2014/147652

**Published:** 2014-11-19

**Authors:** Tshering Dolma, Reena Mukherjee, B. K. Pati, U. K. De

**Affiliations:** Division of Medicine, Indian Veterinary Research Institute, Izatnagar, Uttar Pradesh 243122, India

## Abstract

The purpose of the study was to determine the immunotherapeutic effect of astaxanthin (AX) on total clinical score (TCS), C-reactive protein (CRP), and neutrophil : lymphocyte ratio in mice mastitis model challenged with pathogenic *Staphylococcus aureus*. Twenty-four lactating mice were divided in 4 equal groups: group I mice served as normal healthy control, group II, positive control, were challenged with pathogenic *S. aureus*, group III mice were challenged and treated with AX, and group IV were treated with amoxicillin plus sulbactum. The TCS was higher in postchallenged mice; however it was significantly higher in group II untreated mice as compared to group III and group IV mice. The neutrophil was higher and lymphocyte count was lower in group II mice at 120 hrs post challenge (PC). The CRP was positive in all the challenged group at 24 hrs PC, but it remained positive till 120 hrs PC in group II. The parameters are related to enhancement of the mammary defense and reduction of inflammation. Hence AX may be used alone or as an adjunct therapy with antibiotic for amelioration of mastitis. Development of such therapy may be useful to reduce the antibiotic burden in management of intramammary infection.

## 1. Introduction

Bovine mastitis is the inflammation of the mammary gland frequently resulting from* Staphylococcus aureus *colonization in the mammary parenchyma causing high economic losses despite intensive research and preventive measures [1].* In vitro* antibacterial sensitivity of commonly used antibioticswas demonstrated by several researchers for* S. aureus *isolates [2]; however the pathogen remains difficult to eradicate with the available antibiotics. Failure in treatment could be due to nonavailability or weak penetration of the antibiotics as the organism survives intracellularly. The greatest demerits of antibiotic treatment are the development of multiple drug resistant bacterial strains and residues in the milk which possess human health hazard. World Health Organization [3] emphasizes need of judicial use of antimicrobials to combat antimicrobial resistance and also to encourage the development of novel preventive and therapeutic aids. Hence search is going on globally for an alternative to antibiotic, or to reduce its dose and duration for therapy. Astaxanthin (AX) is a xanthophyll carotenoid, predominantly of marine origin and naturally obtained from the chlorophyte algae* Haematococcus pluvialis* [4]. Astaxanthin is a highly potent antioxidant apart from it, it anti-inflammatory, immunomodulatory and antibacterial activities [5–8]. Therefore, the present study was undertaken to determine the effect of AX on acute phase protein, neutrophils : lymphocyte ratio, histopathological changes, and clinical recovery in murine staphylococcal mastitis model.

## 2. Materials and Methods

### 2.1. Antibiotic Sensitivity Test (ABST) and Minimum Inhibitory Concentration (MIC) of AX

Astaxanthin was procured from a reputed pharmaceutical company (Zenith Pharmaceuticals, Bangalore, India). Ten mg powder was reconstituted in 10 mL sterile saline solution (NSS), filtered through membrane filter (0.22 *μ*m pore size), and stored in sterile vials. ABST of astaxanthin was done by the disc diffusion method as described by NCCLS [9] against pathogenic* Staphylococcus aureus* isolated from mastitis milk samples and the standard reference* S. aureus* strain (MTCC number 96, Microbial Type Culture Collection, Chandigarh, India). The dose of astaxanthin was calculated by conducting the MIC against* Staphylococcus aureus* by tube dilution method as described in previous papers [10, 11]. For oral drenching in mice, the dosage (in mg/kg) was calculated as per the formulae given below:

(1) $$\begin{array}{c} \text{Dosage}\;\left(\text{mg}/\text{kg}\right)\;\text{of}\;\text{Astaxanthin}=\text{MIC}\times 20. \end{array}$$

### 2.2. Isolation and Characterization of* Staphylococcus aureus*

The dairy cows were screened for mastitis by California Mastitis Test [12]. Isolation and identification of pathogenicorganism from the mastitic milk samples was done as per the standard procedure [13]. The* S. aureus *organism was initially identified on the basis of colony morphology on 5% blood agar as [14] and later by Gram staining and growth on selective media like mannitol salt agar (MSA) and Baird Parker agar plates and further subjected to coagulase test; in brief, 2 test tubes were filled up with 0.5 mL of diluted rabbit plasma; to the first tube 0.1 mL of overnight broth culture of test organism was added and to the second tube 0.1 mL of sterile broth was added and incubated at 37°C for 4 hrs and observed for the coagulation of plasma if positive [14]. Biochemical tests were performed with standard kits (HiStaph Identification Kit, HiMedia, India). However, molecular characterization was not performed. The dose of organism for intramammary challenge was calculated by surface viable count as per the method described elsewhere [15]. Eighteen-hour grown broth culture of* S. aureus* at the rate of 5 × 10^5^ bacteria was taken for challenge in mice in each mammary gland.

### 2.3. Experimental Design and Procurement of Mice

Healthy adult lactating Swiss albino mice weighing around 30–40 grams were procured from the institute's vivarium, after weaning their pups, maintained in the divisional animal shed, and housed in animal cages, providing ad libitum water and feed, under standard temperature, ventilation, and humidity. The experimental trial was conducted in lactating mice using the left fourth (L4) and right fourth (R4) inguinal mammary gland. The mice were grouped into four groups with 6 mice in each group. Group I mice served as healthy negative control. Group II were challenged with* S. aureus* and served as positive control. In group III, astaxanthin was drenched after reconstitution in sterile normal saline solution at the rate of 16 mg/kg body weight from 8 days prior to challenge and continued for 5 days PC orally; group IV was treated with amoxicillin plus sulbactam at the dose rate of 12.5 mg/kg body weight twice daily for 3 days by intramuscular route as depicted in Table 1.

### 2.4. Challenge of Mice with Pathogenic* Staphylococcus aureus*

All the mice of group II, group III, and group IV were inoculated with 18 hrs old culture of* S. aureus* via intramammary route at the rate of 5 × 10^6^ bacteria per teat, under general anesthesia with ketamine at 65 mg/kg and xylazine at 4 mg/kg.

### 2.5. Total Clinical Scores (TCS)

Mammary glands of mice were screened for mastitis by visual examination [16] followed by total clinical score card.

### 2.6. Collection of Blood for Serum Collection and Hematological Parameters

Around 1 mL of blood was collected from the mice using micro capillary tubes on day 0, 24, 72 and 120 hrs post challenge from retrobulbar venous plexus behind the eyeball taking special care not to damage the ocular membrane structure [17]. Serum separation was done for CRP. Simultaneously thin blood smear was made stained with Giemsa stain for differential leukocyte count (DLC) to determine neutrophil : lymphocyte ratio [18].

### 2.7. C-Reactive Protein (CRP)

C-reactive protein was done in serum using a standard kit (CRP kit, Latex Agglutination Method, Span Diagnostics LTD., Mumbai, India) by the manufacturer's instruction.

### 2.8. Histopathological Examination

The mice from each of the four groups were sacrificed after 120 hours PC using xylazine-ketamine combination as injectable anesthesia. Mammary glands L4 and R4 were carefully removed from the skin flaps using blunt scissors and were spread onto a prelabeled glass slide without any air bubbles or hair and stored in 10% (v/v) formalin and further processed for Hematoxylin and Eosin (H&E) staining to visualize the histopathological changes in the tissue section. Mice of all 4 groups were sacrificed at 120 hrs post challenge. Tissue sections of mammary gland of group I lactating mice revealed normal healthy lactating alveoli with no signs of inflammation. In group II mice multiple focal micro abscess predominated by neutrophil and fibrinous exudates in the mammary parenchyma was observed with complete disruption of mammary cellular details. In mice of group III that received astaxanthin, mammary gland was normal with no inflammatory reaction except at the hypodermic adipose tissue and muscularis junction. In group IV mice, the mammary gland tissue section showed relatively less pathological changes as compared to group II. Cellular inflammatory infiltration was mild.

### 2.9. Statistical Analysis

The data collected on each parameter was analyzed by standard statistical methods. Level of significance was set (*P* < 0.05) by applying Friedman test for total clinical scores. Level of significance was set (*P* < 0.05) by applying two-way ANOVA for DLC, using statistical software SPSS (Version 17).

## 3. Results

### 3.1. ABST and MIC of Astaxanthin

The zone of inhibition against isolated* S. aureus* was 26 mm for astaxanthin, whereas it was 32 mm for amoxicillin. The MIC of astaxanthin was 750 *μ*g against isolated* S. aureus.*

#### 3.1.1. Isolation and Biochemical Characterization of* S. aureus*

The Gram staining revealed characteristic Gram positive cocci arranged in small bunches (Staphylo). Confirmation was carried out by performing biochemical test for* Staphylococcus aureus.* Organisms showing positive for catalase, maltose fermentation, coagulase positive, haemolysis of blood agar, oxidation fermentation, methyl red and Voges-Proskauer test were confirmed as* S. aureus.*

### 3.2. Total Clinical Scores (TCS)

There was no variation in TCS at 0 hr, 24 hrs, 48 hrs, 72 hrs, and 120 hrs of observational period in group I mice, whereas in group II, mild inflammation of the mammary glands was observed at 24 hrs PC. At 48 hrs PC in group II mice showed profound depression with appreciable swelling, enlargement, and reddish discoloration with extravasations of blood stained exudates; at 72 and 120 hrs mortality was 60%. On the contrary in group III treated mice there were little inflammatory changes at 48 and 72 hrs PC, with signs of recovery at 120 hrs PC; similarly mild inflammatory changes in mammary gland could be observed at 48 and 72 hrs PC in group IV mice with recovery at 120 hrs PC (Table 2).

### 3.3. DLC in Blood

**Figure 1:**
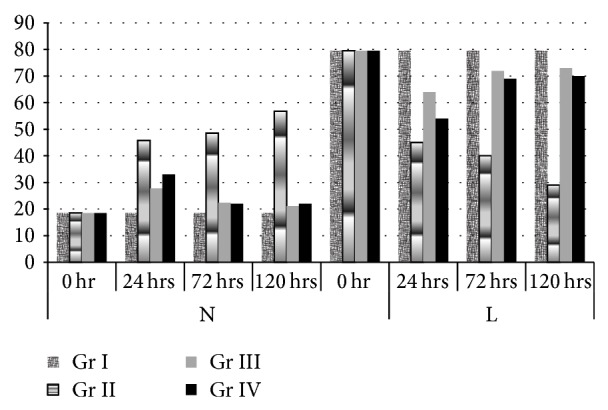
Percent neutrophil and lymphocyte in blood in response to the treatment with astaxanthin (group III) and amoxicillin plus sulbactam (group IV) and in positive control (group II) and in normal healthy mice (group I) (mean ± SE).

DLC was done in all groups at 0 hr, 24 hrs, 48 h, 72 hrs, and 120 hrs post challenge, respectively. At 0 hr lymphocyte count was more and neutrophils count was lesser in all the 4 groups, whereas neutrophil count increased and lymphocyte count decreased significantly in all the infected groups (group II, group III, and group IV) as compared to healthy group (group I) at 24 hrs PC. In group II animals the mean neutrophil count increased to an extent of 66.07% at 120 hrs PC as compared to 0 hr count. Similarly in group III and group IV significantly higher count to an extent of 48.6% and 40.6% could be observed at 24 hrs, respectively; however the neutrophil count at 48 h, 72 hrs, and 120 hrs PC did not differ significantly, whereas the lymphocyte counts in group II animals decreased to an extent of 63% at 120 hrs PC as compared to 0 hr count. Similarly in group III and group IV significantly lower count to an extent of 18.9% and 31.6% could be observed at 24 hrs, respectively; however the lymphocyte count at 48 hrs, 72 hrs, and 120 hrs PC did not differ significantly (Figure 1).

### 3.4. CRP in Serum

The CRP was estimated in serum. On day 0 and 120 hrs post challenge the CRP was negative in all groups. Positive CRP was observed in group II at 24, 48, and 76 hrs PC, whereas in group III the CRP positive reaction was recorded at 24 hrs PC only, whilst the CRP reaction was negative in group IV at 24 hrs PC.

### 3.5. Histopathological Examination

Mice were sacrificed at 120 hrs PC forhistopathological examination of mammary tissue. Tissue sections of mammary gland of group I mice revealed normal healthy lactating alveoli with no signs of inflammation. In group II mice multiple focal micro abscess predominated by neutrophil and fibrinous exudates in the mammary parenchyma was seen with complete disruption of mammary cellular details. In group III receiving astaxanthin, there were few inflammatory cells with no exudation and the tissue structure was normal. Similarly in group IV cellular inflammatory infiltration was mild and mammary parenchyma appeared normal.

## 4. Discussion

So far antibiotics are the only proven method for the treatment of mastitis; however, antibiotic therapy has got several demerits including the harmful drug residue in food chain. The clinical efficacy of the nonantibiotic agents is recently being researched extensively with promising results; these include bacterial enzymes, antimicrobial peptides, bioresponse modifiers, cytokines, micronutrients, vitamins, and medicinal herbs [18, 19].

Astaxanthin is a xanthophyll carotenoid, naturally obtained from the chlorophyte algae* Haematococcus pluvialis* [4]. In the present trial the immunotherapeutic effect of astaxanthin (AX) was studied in mice staphylococcal mastitis. In the astaxanthin treated mice, the clinical score was almost normal; in the tissue sections of mammary gland the cellular details were almost normal with negligible infiltration of inflammatory cell and little exudation in H&E stained section. AX treatment also resisted the intramammary challenge of pathogenic* S. aureus*; hence no pathological changes could be observed and clinical recovery was 100%. In our experiment AX revealed 26 mm zone of inhibition against isolated* S. aureus*. Kumari and Ramanujan [8] also demonstrated the antibacterial activity of AX against* S. aureus*. The CRP is an acute phase protein and is expressed in early stages of infection, acute phase response was higher in post challenge nontreated mice till 72 hrs PC, whereas the CRP was lower in AX treated mice at 48 and 72 hrs PC; it could be due to the anti-inflammatory potential of AX. Astaxanthin is a potent antioxidant and powerful scavenger of free radicals like superoxide and signet oxygen [20]. It suppresses varied inflammatory mediators like TNF-*α*, IL-1*β*, cyclooxygenase-2, and nitric oxide synthase [21, 22]. In the present study the neutrophil count in post challenge nontreated mice was significantly higher, whereas it significantly decreased after 48 hrs PC in AX treated mice. The AX treatment also increased the lymphocyte count; it indicates the immunomodulatory potential of AX. Kurihara et al. [7] demonstrated the immunomodulatory property of astaxanthin; the authors suggested that astaxanthin treatment in diseased mice inhibited the suppressor of NK cell activity and lipid peroxidation.

## 5. Conclusion

In conclusion, this study represents an initial investigation on the therapeutic use of astaxanthin in mice mastitis model challenged with highly pathogenic* S. aureus*. The results of the present study indicate the antibacterial, anti-inflammatory, and immunomodulatory activity of AX treatment against intramammary challenge with pathogenic bacteria. AX treatment significantly reduced the neutrophil count and acute phase protein and enhanced the lymphocyte count and improved early clinical recovery with no pathological changes in the mammary parenchyma. The present drug trial determines the potential benefits of the AX therapy in intramammary infection in mice model as well as standardization of nonantibiotic agent to reduce antibiotic residue from food chain.

## Figures and Tables

**Table 1:** Therapeutic plan of astaxanthin (AX) in lactating mice challenged with pathogenic *Staphylococcus aureus*.

Groups	Number of mice	Treatment	Dose	Frequency	Route	Interval
Group I	6	Normal healthy mice	—	—	—	—
Group II	6	Positive control	—	—	—	—
Group III	6	AX^*^	16 mg/kg body weight	Twice daily	Orally	Oral drenching of AX for 5 days PC twice a day
Group IV	6	Antibiotic^**^	12.5 mg/kg body weight	Twice daily	Intramuscular	5 days post challenge/twice a day

AX^*^ was drenched orally after reconstitution in sterile normal saline solution, at the rate of 16 mg/kg body weight 10 days prior to challenge and continued 5 days post challenge.

^**^Amoxicillin + sulbactam—via intramuscular route, at 12.5 mg/kg body weight.

**Table 2:** Clinical score card of mice at 0 hr, 24 hrs, 48 hrs, 72 hrs, and 120 hrs post challenge in posttreated mice (mean ± SE).

Groups	0 hr	24 hrs	48 hrs	72 hrs	120 hrs
Group I	0 ± .00^a,x^	0 ± .00^a,x^	0 ± .00^a,x^	0 ± .00^a,x^	0 ± .00^a,x^
Group II	0 ± .00^a,x^	2 ± .00^b,z^	2.5 ± .25^b,y^	3.25 ± .25^c,z^	3 ± .00^c,z^
Group III	0 ± .00^a,x^	1.25 ± .25^b,y^	1.25 ± .25^b,y^	1 ± .25^b,y^	.9 ± .00^a,y^
Group IV	0 ± .00^a,x^	1.25 ± .00^b,y^	1 ± .00^a,y^	1 ± .00^a,y^	.9 ± .00^a,y^

^*^Mean values with dissimilar superscripts in the row (a, b, and c) and column (x, y, and z) vary significantly at *P* < 0.05.
